# Supplementary material for: Yiai Fuzheng decoction inhibits triple-negative breast cancer by remodeling the immune microenvironment
Source: Front Immunol. 2025 Sep 30;16:1615631. doi: 10.3389/fimmu.2025.1615631 (PMC12518410; doi:10.3389/fimmu.2025.1615631)
Supplement: Supplementary file 2 [file DataSheet1.zip › Supplementary File 2/Treatment/C_vs_D_vs_H/06_Pathway_Analysis/Pathway_Analysis/Sig_Pathways_ID/Alanine, aspartate and glutamate metabolism.pdf]

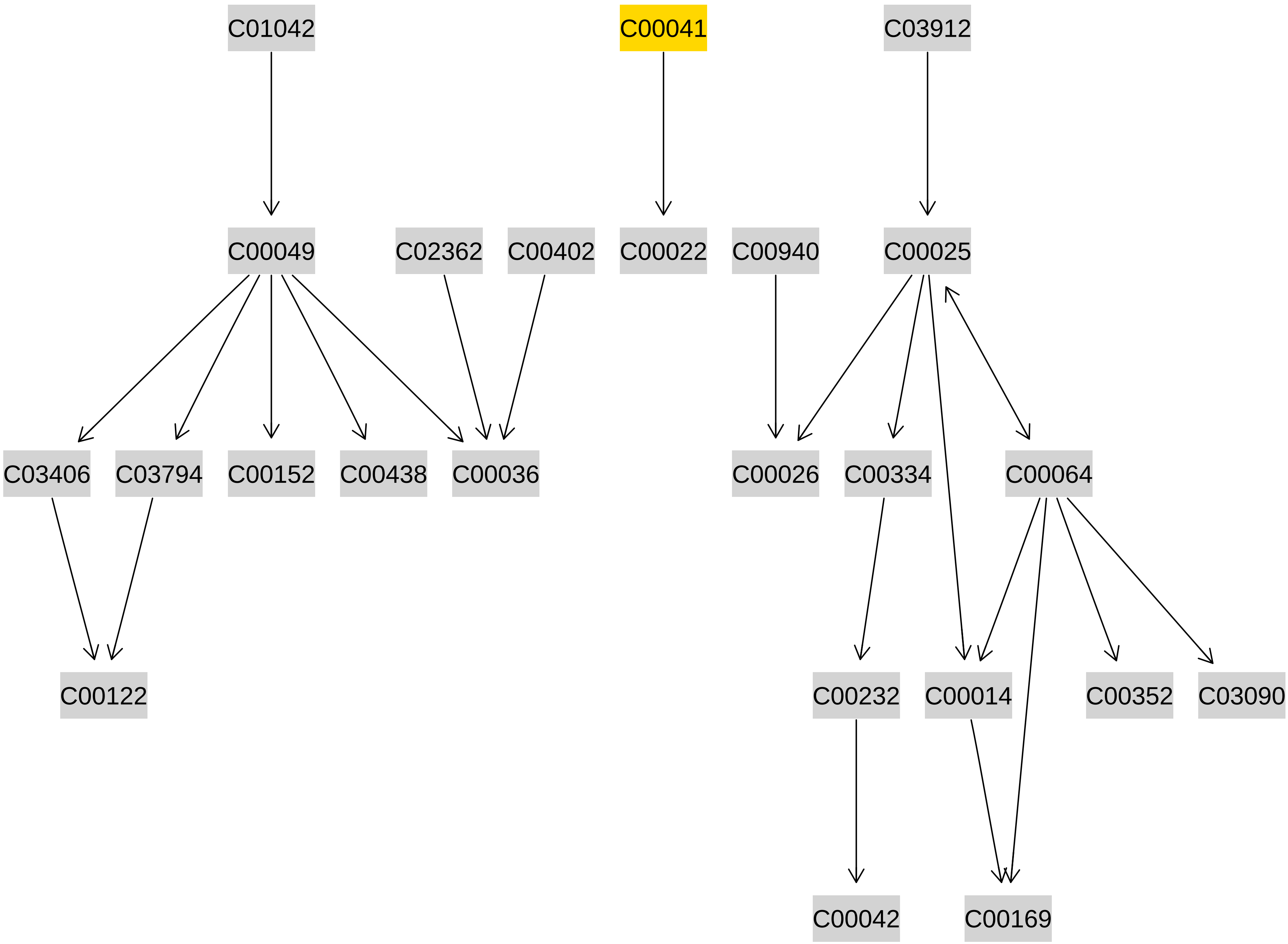
C01042
C00041
C03912
C00049
C02362
C00402
C00022
C00940
C00025
C03406
C03794
C00152
C00438
C00036
C00026
C00334
C00064
C00122
C00232
C00014
C00352
C03090
C00042
C00169
